# MAF-Net: Multimodal cross-attention-based fusion network for cardiovascular disease classification

**DOI:** 10.1371/journal.pone.0345238

**Published:** 2026-04-07

**Authors:** Chang Qu, Xin Zhang, Yansong Lu, Yi Wang, Chengzhi Su

**Affiliations:** School of Artificial Intelligence, Changchun University of Science and Technology, Changchun, Jilin, China; South China University of Technology, CHINA

## Abstract

Cardiovascular disease ranks among the leading causes of death globally, posing a severe threat to human health. Consequently, rapid and accurate identification of cardiovascular disease has become a critical research endeavor. Electrocardiograms (ECGs), as a non-invasive detection tool, are widely used in cardiovascular disease detection due to their convenience and effectiveness. However, existing methods are often limited to single-modality analysis, neglecting the interaction between clinical data (such as age, gender, weight, etc.) and ECG features in classification tasks, resulting in limited recognition accuracy. Integrating multimodal data is key to improving CVD diagnostic accuracy. To address this, we propose MAF-Net (Multimodal Cross-Attention-based Fusion Network), a multi-class classification model that fuses clinical data features with ECG signal features for cardiovascular disease classification. The model comprises three components: (1) X Branch (Clinical Data Processing): Generates high-order interaction features via a second-order polynomial feature cross-layer and employs channel attention-weighted selection to identify key clinical factors;(2) Y Branch (Multi-scale ECG Feature Extraction): Parallel multi-scale convolutional modules (64@7, 128@3, 256@3) capture local morphological features, while Bi-LSTM models long-range temporal dependencies, supplemented by multi-head attention to focus on pathological segments;(3) Bidirectional Modality Fusion Module: Employing a bidirectional cross-attention mechanism, it uses clinical features as Query and ECG features as Key/Value to deeply fuse clinical and ECG data features. On the dataset, experiments targeting five super-categories of arrhythmias— NORM (Normal), MI (Myocardial Infarction), STTC (ST-T Segment Changes), CD (Conduction Disturbance), HYP (Hypertrophy). showed an accuracy rate of 90.75% ± 0.32%, precision of 84.58% ± 0.41%, and recall of 87.12% ± 0.38%, with an F1 score of 0.8069 ± 0.005 and a ROC-AUC value of 0.9407 ± 0.002. Results indicate that this model outperforms existing methods across key metrics, demonstrating its potential for application in clinical decision support.

## 1. Introduction

According to the World Health Organization (WHO), cardiovascular disease (CVD) remains the leading cause of death globally, accounting for 17.9 million deaths in 2019 (32% of all global deaths) [1]. Early diagnosis is crucial for risk reduction and can be effectively managed through medication and interventions. Research indicates that patient clinical information (e.g., age, gender, weight) is closely associated with CVD and serves as important risk predictors [2,3].

As a key non-invasive diagnostic tool in cardiology, electrocardiography (ECG) captures extensive physiological and pathological cardiac activity, enabling assessment of a patient’s clinical cardiac status for CVD diagnosis [4,5].In traditional CVD diagnosis, physicians typically rely on patient history and clinical examinations, using a set of quantified medical parameters to classify patients into CVD disease categories. However, this approach is inefficient when handling complex and diverse data, and the limited number of experienced cardiovascular specialists can lead to misdiagnosis [6]. Currently, machine learning is widely applied in medicine. Techniques such as Support Vector Machines (SVM) [7,8], Random Forests (RF) [9], and Dynamic Bayesian Networks (DBN) [10] have been employed by experts for cardiovascular disease diagnosis support. However, most of these machine learning algorithms rely heavily on complex data preprocessing and cumbersome manual feature selection [11,12], hindering their clinical application in cardiovascular disease.

Recently, deep learning (DL) has demonstrated superior performance across numerous medical tasks [13]. [14] et al. proposed a novel approach for classifying similar physiological signals with periodic characteristics. By leveraging a CNN-LSTM network, they constructed temporal representation inputs that integrate ECG periodicity features, outperforming traditional feature fusion methods and advancing innovative diagnostic approaches for CVD. [15] et al. combined Butterworth bandpass filtering with discrete wavelet transform (DWT) to reduce noise and enhance signals when classifying heart diseases using ECG signals. This approach yielded clearer signals and improved the accuracy of extracted morphological features. [16] et al. employed a cross-attention-based transformer model to efficiently extract embedded ECG features. This approach enables processing of data sequences with varying lengths and complexities, yielding meaningful embedded features for more accurate results. Experimental results show the proposed method achieved a challenge score of 0.6112, outperforming other approaches. However, most current AI models are limited to single-modality ECG analysis, neglecting clinically relevant data with clear prognostic value, resulting in diagnostic ceilings that are difficult to overcome. These models suffer from severe overfitting on small samples and limitations in handling single-modality data within cardiovascular disease diagnosis.

Most existing AI-based ECG classification methods rely on extracting and analyzing ECG signal features. While these approaches effectively utilize information-rich data within ECG signals, patient-related clinical features such as age, gender, and medical history are often overlooked [17].Due to distributional differences between patient clinical data and ECG data, the normalization process risks obscuring the unique characteristics of different data types, potentially weakening key features of one category. When using a common classification model for multi-modal data, differing feature expression requirements across modalities may cause underfitting, leading to degraded classification performance [18].

Regarding feature fusion, [19] et al. categorized fusion strategies into three types—early fusion, late fusion, and joint fusion—addressing multimodal data integration at the feature, output, and model levels to enhance multimodal learning effectiveness.By employing various fusion methods to integrate clinical and imaging data, [20] not only generated a context-aware model that reduced misdiagnosis rates and diagnostic delays but also informed future work by exploring optimal data selection and fusion strategies. [21] Employing two independent feature extraction models to explore intermediate and late fusion strategies effectively combines data from different modalities, enhancing cardiovascular disease detection capabilities. However, these fusion methods merely perform simple feature concatenation, resulting in fused features lacking sufficient representativeness.

To address these limitations, we propose the multimodal cross-attention fusion network model MAF-Net. Feature Extraction Stage: A fully connected neural network (FCNN) extracts features from clinical data to capture patterns in structured information. Multiscale convolutional neural networks (CNNs) combined with bidirectional long short-term memory (Bi-LSTM) and multi-head attention mechanisms process ECG data to effectively handle temporal signals. Feature Interaction Fusion Stage: Bidirectional interaction between multi-scale CNN-extracted temporal signal features and FCNN-extracted structured data features is achieved through cross-attention mechanisms. Feature concatenation strategies fuse these two feature types to preserve and leverage their complementary information. Dual-Branch Network Design: An independent dual-branch network architecture prevents cross-interference between heterogeneous data, thereby enhancing feature purity and model generalization capability.

This study aims to address the aforementioned research gaps with the following specific objectives:

(1) Propose a novel MAF-Net model to achieve deep fusion of clinical data and ECG signals, surpassing the performance of single-modality models.
(2) Design a bidirectional cross-attention mechanism to dynamically interact with both modalities, moving beyond simple feature concatenation.
(3) Validate model performance on the publicly available PTB-XL dataset, demonstrating superiority through comprehensive ablation studies and comparative analyses.

The structure of this paper is as follows: Section 1 describes the materials, Section 2 introduces the methods, Section 3 presents the model’s various test performance results on the PTB-XL dataset, Section 4 discusses the experimental findings, and Section 5 concludes.

## 2. Materials

### 2.1 Dataset and data preprocessing

This study utilizes the publicly available PTB-XL dataset, which contains de-identified clinical electrocardiogram (ECG) recordings. All data were anonymized prior to release and did not require Institutional Review Board approval. The dataset originates from the open-source PTB-XL ECG dataset published on PhysioNet [22] in 2020.The PTB-XL ECG dataset is a large public dataset comprising 21,837 clinical 12-lead ECGs from 18,885 patients, each lasting 10 seconds. Additionally, the dataset includes extensive clinical data supplemented by demographic information (e.g., gender, age, weight), infarct characteristics, the likelihood of diagnostic ECG statements, and annotations of signal properties.The dataset provides raw waveform recordings at 500 Hz and 100 Hz sampling rates. These raw waveform data are annotated by up to two cardiologists and structured as a multi- class dataset, with diagnostic statements aggregated into five superclasses as shown in Table 1, which also includes the number of records classed for each disease.

**Table 1:** Number of records classed for five superclass disease categories in the PTB-XL dataset.

Records	Superclass	Description
9528	NORM	Normal ECG
5486	MI	Myocardial Infarction
5250	STTC	ST/T Change
4907	CD	Conduction Disturbance
2655	HYP	Hypertrophy

During the data preprocessing stage, due to the low amplitude of ECG signals, they are highly susceptible to interference from other factors, which can compromise detection accuracy. Therefore, denoising preprocessing is essential. This study employs a wavelet threshold denoising method for signal cleaning and utilizes baseline removal techniques to eliminate baseline drift.

Class Imbalance Handling: To address the class imbalance in the PTB-XL dataset, we employed a weighted cross-entropy loss function during model training. The weight assigned to each class is inversely proportional to its sample count in the training set.

Missing Value Handling: For missing values in clinical data, we employed median imputation for numerical features and mode imputation for categorical features.

Multi-class Handling: Since the PTB-XL dataset contains multi-class records, we employ multi-hot encoding to represent overlapping diagnoses, allowing each sample to belong to multiple disease categories simultaneously. During training, we utilize the sigmoid activation function and binary cross-entropy loss function to address the multi-class classification task.

This study employs 10-fold cross-validation, ensuring data from the same patient does not span multiple folds through stratified sampling. Folds 1–8 are used for training, fold 9 for validation, and fold 10 for testing, excluding empty- class samples. Ultimately, the training, validation, and test sets comprise 17,441, 2,193, and 2,203 samples, respectively.

### 2.2 Experimental setup

All experiments were conducted in the following environment: Python 3.8, TensorFlow 2.9, NVIDIA GeForce RTX 4070 SUPER GPU.To ensure reproducibility, the random seed was fixed at 42. Models were trained using the Adam optimizer with an initial learning rate of 1e-4, batch size of 64, and 100 epochs, employing early stopping (Patience = 10) to prevent overfitting.

Hyperparameter selection: Convolution kernel size (7x3x3), LSTM cell size (128), attention heads (4), etc., were determined via grid search combined with 5-fold cross-validation to balance model complexity and performance.

Regularization Methods: In addition to Dropout, the model employs L2 weight decay (coefficient 1e-4) and Batch Normalization (BatchNorm) to prevent overfitting.

## 3. Method

### 3.1 Multimodal cross-attention fusion network MAF-Net

**Fig 1:**
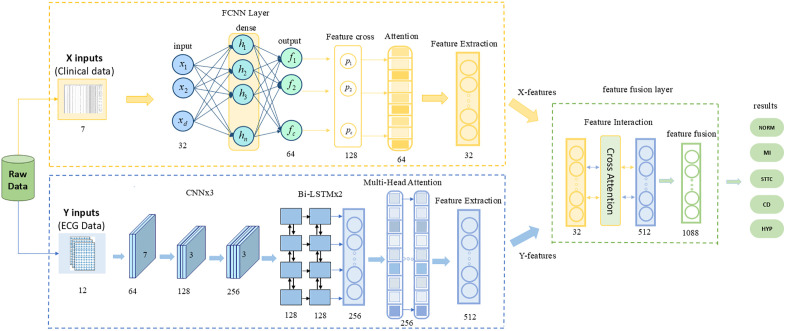
Structural flowchart of the multimodal cross-attention fusion network MAF-Net model.

This paper proposes a multimodal cross-attention fusion network model, MAF-Net, as shown in Fig 1. Its architecture comprises three core components: the X branch, the Y branch, and the fusion module. The X branch processes structured clinical data through deep expansion, feature cross-attention, and feature attention-weighted modules to produce low-dimensional feature representations. The Y branch employs multi-scale CNNs to extract local features from ECG data, combines bidirectional LSTMs to capture temporal information, and utilizes multi-head attention to focus on critical segments, ultimately generating features via global pooling. The fusion module innovatively employs a bidirectional cross-attention mechanism to achieve bidirectional interaction and merging of features from both branches, ultimately outputting classification results. By enhancing feature interaction and information fusion capabilities, this model effectively captures complex correlations between clinical data and ECG signals, significantly improving classification performance while maintaining training stability.

#### 3.1.1 MAF-Net Model X branch.

**Fig 2:**
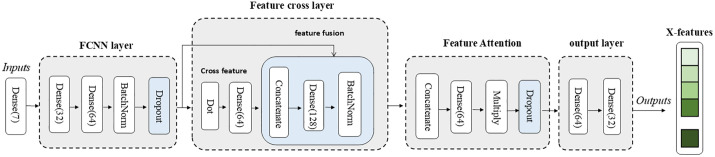
Model structure flowchart of the X branch (clinical data processing branch) in the MAF-Net model.

The input layer (X-inputs) receives structured feature data including patient information and diagnostic classes. Patient clinical data serves as foundational information in diagnosis, aiding in the analysis of medical data’s clinical significance and playing a crucial role in enhancing the accuracy of intelligent diagnosis. The structured clinical data classification model is illustrated in Fig 2.

Branch X consists of three modules: a fully connected neural network (FCNN), a second-order polynomial feature cross-layer, and a feature-level attention mechanism.The depth extension module maps Dense(32) to Dense(64). It employs BatchNorm to achieve mean zeroing and variance normalization, while Dropout (rate = 0.3) randomly discards 30% of neuron outputs to prevent overfitting. This enables higher-order combinations of structured features, capturing complex relationships among clinical characteristics.

The FCNN (Fully Connected Neural Network) comprises Dense, BatchNorm, and Dropout layers, defined by the following formula:


$$h_{\text{1}}\;=Dropout\left(BatchNorm\left(ReLU(W_{\text{1}}\;x+b_{\text{1}})\right),d\right)$$ (1)

Where x is the output feature vector, $W_{\text{1}}$ is the weight matrix of the fully connected layer, $b_{\text{1}}$ is the bias term, d is the dropout rate (0.3), and $h_{\text{1}}$ is the feature vector.

The second-order polynomial feature interaction layer is a network layer designed to generate second-order interaction terms between raw features. It computes pairwise dot products among input features using the Dot layer (Dot) to capture nonlinear relationships between features.

The formula for the Quadratic Polynomial Feature Cross Layer is defined as follows:


$$c_{out}=ReLU(W_{\text{2}}(\frac{h_{\text{1}}\cdot h_{\text{1}}}{{\|h_{\text{1}}\|}_{\text{2}}})+b_{\text{2}})$$ (2)

Here, $h_{\text{1}}\cdot h_{\text{1}}$ denotes the dot product of the feature vector (`Dot(axes = 1, normalize = True)`).

The feature attention module concatenates input features with cross-features, generates attention weights through a Dense(64) layer, and applies these weights to compressed features via a Multiply layer to amplify important feature weights.Subsequently, Dropout (rate = 0.3) randomly discards some neuron outputs to prevent overfitting. The attention-weighted Dense(64) layer is then compressed to a lower-dimensional Dense(32) layer, serving as the model’s final feature representation output. This output is normalized to align closely with the Y branch.

The feature attention formula is as follows:


$$y=ReLU(W_{\text{4}}\;(Dropout(\left(h_{\text{1}}⊙\sigma \left(W_{\text{3}}\;[x,c_{out}\;]+b_{\text{3}})\right),d\right))+b_{\text{4}}\;)$$ (3)

Where σ is the activation function (sigmoid), ⊙ denotes element-wise multiplication (Multiply layer), and Y is the output feature.

Through multi-layer nonlinear transformations, FCNN can automatically extract high-order features from complex patient clinical text data, effectively enhancing the accuracy and efficiency of medical data analysis.

#### 3.1.2 MAF-Net Model Y branch.

**Fig 3:**
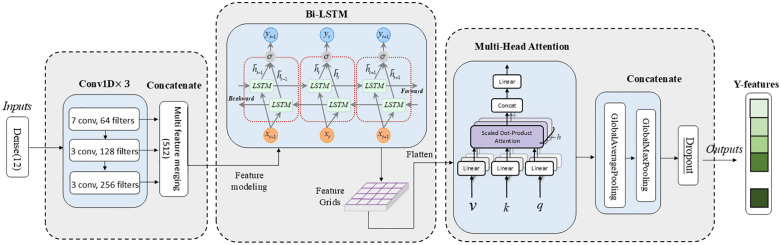
Model structure flowchart of the Y branch (ECG data processing branch) in the MAF-Net model.

The input layer (Y-inputs) receives ECG data processed through data preprocessing. As shown in Fig 3, the model structure comprises four components: a multi-scale CNN module, a bidirectional LSTM module, a multi-head attention mechanism module, and a global pooling module.

Within the multi-scale CNN module, a three-layer convolutional neural network processes ECG data in parallel. Convolution kernel sizes of 7 × 3 × 3 are employed to capture waveform features at different temporal scales within the ECG signal (e.g., P waves, QRS complexes, T waves).The first layer employs 64 channels and 7 convolutional kernels with a large receptive field, suitable for extracting low-frequency features (e.g., P wave and T wave morphology) to enhance the model’s expressive power for input data. The second layer uses 128 channels and 3 convolutional kernels with a medium receptive field to extract fine-grained local features, balancing local and global characteristics (e.g., QRS complex detection).The third layer employs 256 channels and 3 convolutional kernels with a small receptive field, focusing on high-frequency details (e.g., R-wave peaks, noise interference). Batch normalization (BatchNorm) is applied across all layers to accelerate training and enhance stability. Subsequently, features extracted at different scales from the three convolutional layers are merged, preserving multi-scale information. This approach overcomes the limitations of single-layer convolutions, satisfies global feature extraction requirements, and is well-suited for steady electrocardiophysiological signals.

**Multiscale CNN Module**: Input signal $X\in {\mathbb{R}}^{T\times d}$ (time step T, feature dimension d) passes through three convolutional layers with distinct kernel sizes.

The multiscale feature fusion principle is formulated as follows:


$$H_{\text{1}}\;=ReLU\left(Conv1D\left(X,W_{\text{1}}\;,k=7\right)\right)\in {\mathbb{R}}^{T\times 64}$$ (4)

$$H_{\text{2}}\;=ReLU\left(Conv1D\left(X,W_{\text{2}}\;,k=3\right)\right)\in {\mathbb{R}}^{T\times 128}$$ (5)

$$H_{\text{3}}\;=ReLU\left(Conv1D\left(X,W_{\text{3}}\;,k=3\right)\right)\in {\mathbb{R}}^{T\times 256}$$ (6)

$W_{\text{1}}\;,W_{\text{2}}\;,W_{\text{3}}\;$, where are the convolutional kernel weights, and the number of output channels are 64, 128, and 256 respectively.

Batch Normalization and Feature Concatenation


$$Hmerged\;=\left[BatchNorm\left(H_{\text{1}}\;\right),BatchNorm\left(H_{\text{2}}\;\right),BatchNorm\left(H_{\text{3}}\;\right)\right]\in {\mathbb{R}}^{T\times (64+128+256)}$$ (7)

Bi-LSTM consists of two independent LSTMs: Forward LSTM: Analyzes waveform evolution over time (e.g., P → QRS → T) Backward LSTM: Captures reverse dependencies (e.g., T-wave changes may indicate preceding abnormalities). Utilizes temporal pattern learning: Better understands the time-series characteristics of signals, captures long-term dependencies in ECG signals, and preserves the temporal dimension.The 128-dimensional forward + 128-dimensional backward features from the bidirectional LSTM are compressed into a 128-dimensional temporal attention feature.

**Bidirectional LSTM Module**: Inputs multi-scale features ($H_{merged}$) to extract temporal dependencies via bidirectional LSTMs.

Forward and backward LSTM


$$\vec{h_{t}}=LSTM\left(\vec{h_{t-1}},H_{merged},t\right)\in {\mathbb{R}}^{128}$$ (8)

$$\overset{\leftarrow }{h_{t}}=LSTM\left(\overset{\leftarrow }{h_{t+1}},H_{merged},t\right)\in {\mathbb{R}}^{128}$$ (9)

Bidirectional Output Concatenation


$$L_{\text{out}}=\left|\vec{h_{t}},\overset{\leftarrow }{h_{t}}\right|\in {\mathbb{R}}^{T\times 256}$$ (10)

Within the multi-attention mechanism module, input data is segmented into multiple heads, with attention calculated separately for each before merging results. This captures features from different positions and subspaces within the input ECG data. By dynamically focusing on key waveform segments, the model autonomously learns which features are more critical for disease classification, enhancing classification accuracy and robustness in cardiovascular disease identification.

**Multi-head Attention Mechanism Module**: Input: $L_{\text{out}}$ Calculate self-attention and perform residual connection.

The mathematical formula for the Multi-Head Attention mechanism is as follows:


$$head_{i}=Attention\left(L_{out}\;\overset{Q}{\underset{i}{W}}\;,L_{out}\;\overset{K}{\underset{i}{W}}\;,L_{out}\;\overset{V}{\underset{i}{W}}\;\right)\;$$ (11)

$$A_{\text{out}}=Concat(head_{\text{1}}\;,\ldots ,head_{\text{4}}\;)W^{O}\in {\mathbb{R}}^{T\times 256}$$ (12)

Where num_heads = 4 and head_dim = 32 per head. $\overset{Q}{\underset{\text{i}}{W}}\;,\overset{K}{\underset{i}{W}}\;,\overset{V}{\underset{i}{W}}\in {\mathbb{R}}^{256\times 32}$, $W^{O}\in {\mathbb{R}}^{128\times 256}$, $\overset{Q}{\underset{i}{W}}$, $\overset{K}{\underset{i}{W}}$, and $\overset{V}{\underset{i}{W}}$ are learnable parameter matrices used to project Q, K, and V into different subspaces. $W^{O}$ is a linear transformation matrix applied after multi-head attention to convert the concatenated results to the desired dimension.

Layer Normalization (Residual Connection)


$$A_{norm}\;=LayerNorm(L_{out}\;+A_{out}\;)$$ (13)

In the feature extraction module of, global average pooling is applied to the temporal dimension to extract local features, while global max pooling is used on the temporal dimension to extract the most salient features. Features from both pooling methods are concatenated to produce a fixed-length feature vector. Finally, a Dropout layer randomly discards some output neurons to prevent overfitting, yielding the extracted features for the Y branch.

**Global Pooling Module**: Pools the attention outputs $A_{norm}\in {\mathbb{R}}^{T\times 256}$.

Global Average Pooling (GAP) formula:


$$g_{avg}=\frac{\text{1}}{T}\sum_{t=1}^{T}A_{norm},t\in {\mathbb{R}}^{256}$$ (14)

Global Max Pooling (GMP) is expressed as:


$$g_{max}={max}_{t}A_{norm},t\in {\mathbb{R}}^{256}$$ (15)

Pooling Result Concatenation


$$y_{features}\;=\left[g_{avg}\;,g_{max}\;\right]\in {\mathbb{R}}^{512}$$ (16)

#### 3.1.3 Feature fusion module of the MAF-Net model.

**Fig 4:**
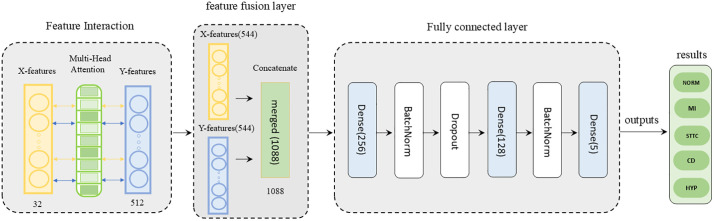
Feature fusion model structure flowchart for the X branch (clinical data) and Y branch (ECG data) in the MAF-Net model.

The feature fusion model architecture is illustrated in Fig 4. This structure comprises three components: First, within the bidirectional feature interaction module, a multi-head attention mechanism layer enables bidirectional interaction between X-branch and Y-branch features. Subsequently, in the feature fusion module, a Concatenate layer further combines the augmented X-branch and Y-branch features to form the final feature representation, preserving information from both input streams.Finally, within the fully connected layer module, two fully connected layers process the merged features to reduce dimensionality and extract a more compact feature representation. The final output is generated using a Sigmoid activation function in the last fully connected layer, completing the classification task.

Bidirectional Cross-Attention Mechanism: Unlike the unidirectional cross-attention in traditional Transformers, this paper’s bidirectional cross-attention mechanism simultaneously performs interactions in both directions: X → Y and Y → X. This enables clinical data to guide ECG feature interpretation while ECG features can also refine clinical data judgments, achieving deeper modality fusion.

Feature Cross-Attention

Bidirectional feature interaction is implemented using MultiHeadAttention in the code, as described by the following formula:

Feature Input Expansion: The original features x (from the X branch) and y (from the Y branch) are reshaped into sequence form.


$$X^{\prime }\in {\mathbb{R}}^{1\times d_{x}},\text{\textbackslash{}hspace\{0.17em}\;\}Y^{\prime }\in {\mathbb{R}}^{1\times d_{y}}$$ (17)

Where $d_{x}$ and $d_{y}$ represent the dimensions of x_features and y_features, respectively.

Multi-head attention computation: Query-Key-Value projection (using x → y as an example).


$$Q_{x}\;=W_{Q}\;x\prime ,\text{\textbackslash{}hspace\{0.17em}\;\}K_{y}=W_{K}\;y\prime ,\text{\textbackslash{}hspace\{0.17em}\;\}V_{y}\;=W_{V}\;y\prime$$ (18)

Here, $W_{Q}\;,W_{K}\;,W_{V}\;$ is a learnable weight matrix.

Attention weights (Scaled Dot-Product Attention) are calculated using the formula:


$$Attention\left(Q_{x},K_{y},V_{y}\right)=soft\;max\left(\frac{Q_{x}\overset{T}{\underset{y}{K}}}{\sqrt{{\text{d}}_{k}}}V_{y}\right)$$ (19)

Where $Q_{X}\overset{T}{\underset{y}{K}}$ calculates the similarity between queries and keys. $\sqrt{d_{k}}$ is the scaling factor to prevent vanishing gradients from excessively large dot products. softmax converts similarity into a probability distribution (weights). The final output is obtained through weighted summation (weighted ×$V_{y}$).

Multi-head Output Concatenation:


$$x_{to}\_y\;=Concat(head_{\text{1}}\;,\ldots ,head_{\text{4}})W_{O}\;$$ (20)

Where $W_{O}$ is the output projection matrix.

Bidirectional Cross-Attention


$$x_{to\_y}\;=MultiHeadAttention(x\prime ,y\prime ,y\prime )$$ (21)

$$y_{to\_x}\;=MultiHeadAttention(y\prime ,x\prime ,x\prime )$$ (22)

**Feature Fusion**: Enhanced Feature Fusion: Merge features after bidirectional enhancement.


$$x_{enhanced}\;=\left[x,y_{to\_x}\;\right]\in {\mathbb{R}}^{d_{\text{x}}\;+d_{y\_to\_x}\;}$$ (23)

$$y_{enhanced}\;=\left[y,x_{to\_x}\;\right]\in {\mathbb{R}}^{d_{y}\;+d_{x\_to\_y}\;}$$ (24)

Global Feature Fusion: Merge features after bidirectional enhancement.


$$z=\left[x_{enhanced}\;,y_{enhanced}\;\right]\in {\mathbb{R}}^{\left(d_{\text{x}}\;+d_{y\_to\_x}\;)+(d_{y}\;+d_{x\_to\_y}\;\right)}$$ (25)

Classification Head Calculation: Output prediction results through a fully connected layer.


$${\text{h}}_{\text{1}}=ReLU(W_{\text{1}}\;z+b_{\text{1}}\;)(dim=256)$$ (26)

$$h_{\text{2}}=ReLU(W_{\text{2}}\;h_{\text{1}}\;+b_{\text{2}}\;)(dim=128)$$ (27)

$$o=\sigma \left(W_{\text{3}}\;h_{\text{2}}\;+b_{\text{3}}\;\right)\left(dim=Z\_shape\left[-1\right]\right)$$ (28)

Here, σ denotes the sigmoid activation function, which constrains output values between 0 and 1 for multi- class classification tasks. In ECG classification, each output node corresponds to a cardiovascular disease (CVD), and the sigmoid activation function outputs an independent probability value for each disease category.

### 3.2 Performance metrics

To evaluate model performance, we validate the MAF-Net model on the PTB-XL dataset. Four key performance metrics are used to assess model effectiveness: accuracy, precision, recall, and F1 score. Additionally, the area under the curve (AUC) value is calculated: These metrics are crucial for evaluating the reliability and efficiency of diagnoses in clinical settings. They are defined as follows:


$$Accuracy=\frac{TP+TN}{TP+TN+FN+FN}$$ (29)

$$precision=\frac{TP}{TP+FP}$$ (30)

$$Recall=\frac{TP}{TP+FN}$$ (31)

$$F1-score=\frac{2Precision\cdot Recall}{Precision+Recall}$$ (32)

TP Indicates true positives: the number of samples correctly predicted by the model to be in the positive category;

TN Indicates true negatives: the number of samples correctly predicted by the model to be in the negative category;

FP denotes false positive: the number of samples that the model incorrectly predicts as a positive category;

FN denotes false negative: the number of samples that the model incorrectly predicts as negative classes.

To comprehensively evaluate classifier performance, we constructed ROC curve area plots, confusion matrices, bar charts, and spatial clustering maps to visualize classification results. These methods effectively assessed the classifier’s ability to distinguish cardiovascular disease (CVD) patients from healthy subjects, ensuring a thorough understanding of model performance.

Statistical Significance Testing: We employed a paired t-test to compare performance differences between MAF-Net and baseline models, setting the significance level α = 0.05. All experiments were repeated five times, with results reported as mean ± standard deviation.

## 4. Experimental results

To further validate our proposed method’s performance in multi-class ECG-based CVD diagnosis, we compared it with several recently studied approaches. All comparative experiments were conducted on the identical PTB-XL dataset, and results were based on experimental settings and performance metrics reported in the original literature to ensure fair and consistent evaluation.

### 4.1 Performance comparison with other models

Our proposed MAF-Net achieved competitive results. For completeness of comparison, the baseline model results listed in Table 2 represent the best values reported in the original papers (without variability metrics), while our results are the mean ± standard deviation from 5 independent experiments. To enable fair comparisons, future work will focus on reproducing baseline models under identical experimental settings.We use paired t-tests (significance level α = 0.05) to assess the statistical significance of performance differences.

**Table 2:** Performance metrics comparison between our MAF-Net model and various methods (mean ± standard deviation).

Model	Accuracy	Precision	Recall	F1 Score	ROC-AUC
CIGRU-ELM [23]	89.00%	90.20%	86.80%	0.8800	0.8890
STADSNet [24]	89.11%			0.8152	0.9307
STFAC-ECGNet [25]	89.50%	84.90%	84.00%	0.8440	0.9320
MRF-CNN [26]	89.70%	73.00%	71.00%	0.7200	0.9300
MAF-Net (ours)	**90.75%±** **0.32%**	**84.58% ± 0.41%**	**87.12% ± 0.38%**	**0.8069 ± 0.005**	**0.9407 ± 0.002**

(Performance data for comparison models in the table are directly quoted from their original literature, where standard deviation was not reported. Our MAF-Net results are based on the mean ± standard deviation from 5 random runs. p-value < 0.05 indicates statistical significance).

As shown in Table 2, the multimodal cross-attention fusion network (MAF-Net) achieved high accuracy for cardiovascular disease classification: precision 90.75% ± 0.32%, recall 84.58% ± 0.41%, recall 87.12% ± 0.38%, F1 score 0.8069 ± 0.005,and a macro AUC of 0.9407 ± 0.002. The MAF-Net model achieved the best performance in both accuracy (90.75% ± 0.32%) and AUC (0.9407 ± 0.002).Our approach highlights the enhancement of cardiovascular disease diagnosis performance across the entire classification model by leveraging the auxiliary differences between branches that extract clinical features.

### 4.2 Ablation study results

We conducted ablation experiments to evaluate the effectiveness of each branch within the model, assessing performance under identical conditions: classification performance using only the X-branch network, classification performance using the Y-branch network, and classification performance using the dual-branch fusion model. Table 3 shows the classification performance of the model under these different configurations.

**Table 3:** Ablation experiment results for the MAF-Net model (mean ± standard deviation).

Model	Accuracy	Precision	Recall	F1 Score	ROC-AUC
X (Clinical Data Only)	80.80% ± 0.60%	75.00% ± 0.80%	34.91% ± 1.20%	0.2550 ± 0.010	0.7916 ± 0.006
Y (ECG data only)	88.86% ± 0.34%	80.91% ± 0.43%	72.62% ± 0.48%	0.7650 ± 0.005	0.9374 ± 0.002
**MAF-Net(X + Y)**	**90.75% ±** **0.32%**	**84.58% ± 0.41%**	**87.12% ± 0.38%**	**0.8069 ± 0.005**	**0.9407 ±** **0.002**

In the patient clinical data classifier of Branch X, the disease classification accuracy reached 80.80% ± 0.60%. This relatively low accuracy stems from the fact that patient clinical data merely reflects basic personal information. As supplementary reference data, it cannot deeply reflect specific physiological indicators, necessitating professional examination and analysis using specialized equipment. In the Y-branch ECG data classification model, the branch network solely performs ECG feature extraction, achieving a disease classification accuracy of 88.86% ± 0.34%. In contrast, the MAF-Net model, which fuses features from both the X and Y branches, achieved an accuracy of 90.75% ± 0.32%. Its classification performance surpassed that of the two independent branch models, indicating that the MAF-Net model—which integrates clinical data features from the X branch and ECG data features from the Y branch—delivers superior classification results compared to the standalone Y-branch ECG feature extraction model.

**Fig 5:**
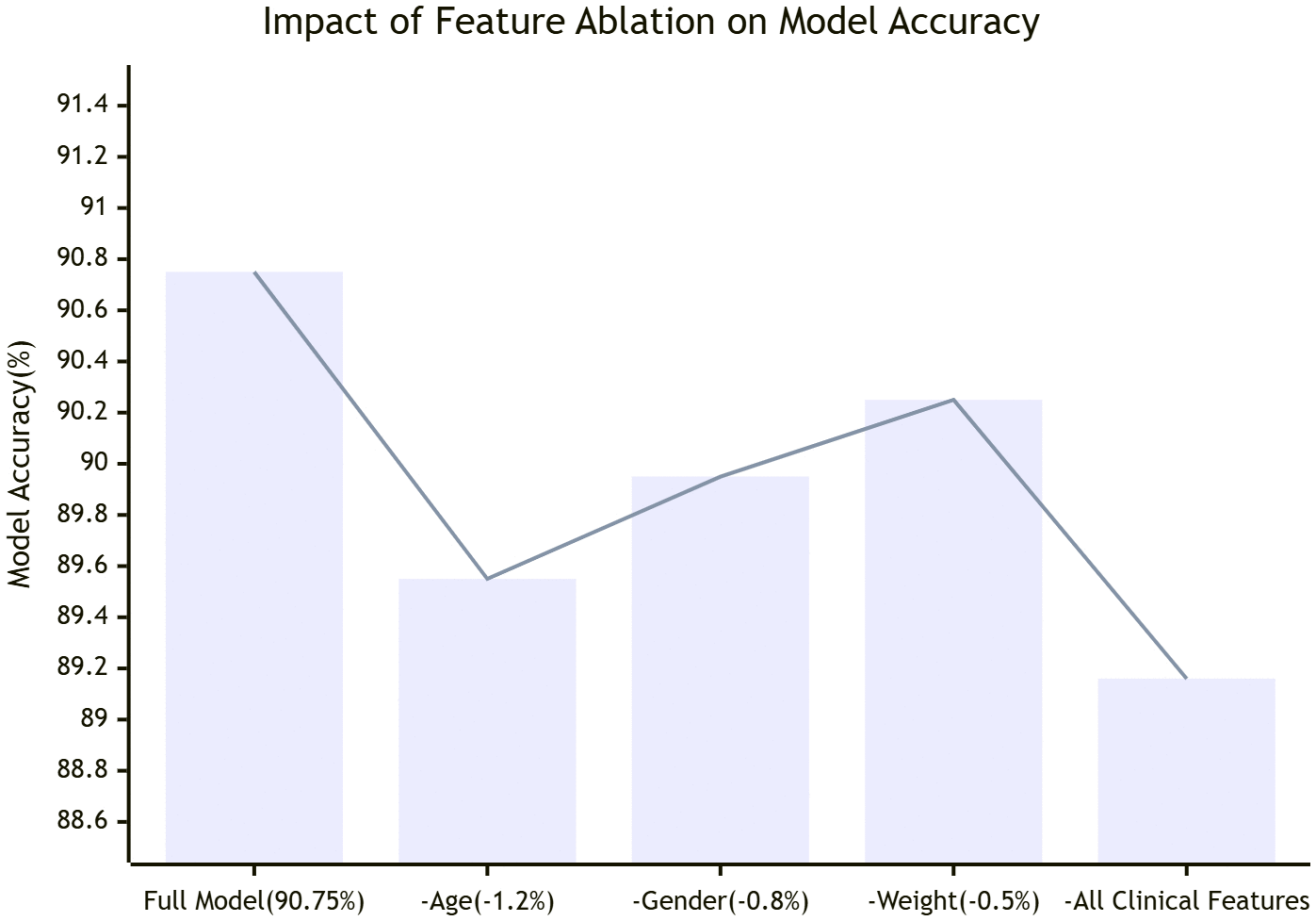
Model recognition accuracy of MAF-Net after removing key clinical features on the PTB-XL dataset.

To further interpret model decisions, we visualized the weights of the cross-attention layer. Feature importance was analyzed by examining the weights of the feature attention module. To investigate the contribution of different clinical features to model performance, we conducted feature ablation experiments, as shown in Fig 5.

As shown in Table 4, After sequentially removing key clinical features such as age, gender, and weight, the model accuracy decreased by approximately 1.2%, 0.8%, and 0.5%, respectively. This indicates that age is the most significant contributing clinical factor, consistent with clinical medical understanding. For instance, in myocardial infarction (MI) cases, the model not only focuses on ST-segment elevation regions in ECG signals but also significantly increases the weight assigned to the “elderly patient history” feature, aligning closely with clinical diagnostic logic. Simultaneously, removing all clinical features reduces model performance to levels comparable to the Y-branch model alone, further validating the effectiveness of multimodal fusion.

**Table 4:** Comparison results of key clinical feature ablation (mean ± standard deviation).

Model	Accuracy
MAF-Net (ECG features only)	88.86% ± 0.34%
MAF-Net (Age Feature Removed)	89.55% ± 0.29%
MAF-Net (Gender Feature Removed)	89.95% ± 0.35%
MAF-Net (with weight feature removal)	90.25% ± 0.31%
MAF-Net	90.75% ± 0.32%

## 5. Discussion

This section examines the impact of the X-branch module on model effectiveness and analyzes model interpretability.

### 5.1 ROC curve comparison

**Fig 6:**
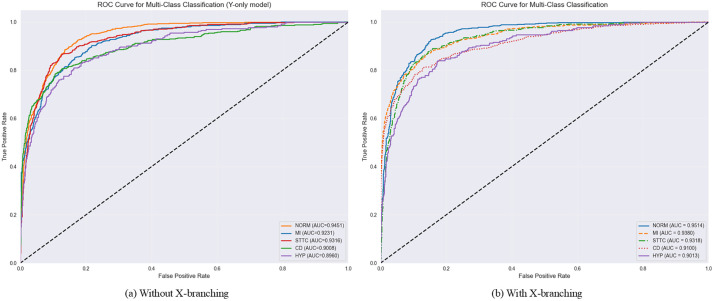
Comparison of ROC curves for the MAF-Net model (with and without X branch) across five hyperclasses on the PTB-XL dataset.

The two ROC curves in Fig 6 are used to evaluate the performance of multi-classification models. The ROC curve reflects the model’s performance at different thresholds by illustrating the relationship between TPR (True Positive Rate) and FPR (False Positive Rate). The AUC value, or the area under the curve, serves as a metric for assessing the model’s overall performance. A value closer to 1 indicates superior model performance.

Fig 6(a) shows the performance of the model without the X branch, covering categories: NORM (Normal), MI (Myocardial Infarction), STTC (ST-T Segment Changes), CD (Conduction Block), HYP (Hypertrophy). The AUC values are 0.9451, 0.9231, 0.9316, 0.9085, and 0.8960 respectively. The model performed well across all categories, particularly in the NORM (normal) category. Fig 6(b) shows the model after incorporating the X branch, which slightly improved AUC values to 0.9514, 0.9380, 0.9318, 0.9100, and 0.9013, notably in the NORM (normal) and MI (myocardial infarction) categories.However, the improvement in the STTC (ST-T segment changes), CD (conduction block), and HYP (ventricular hypertrophy) categories was not significant.Overall, introducing the X branch improved model performance, particularly for NORM (normal) and MI (myocardial infarction) categories. Although the impact on STTC (ST-T segment changes), CD (conduction block), and HYP (ventricular hypertrophy) was limited, the overall performance enhancement indicates that the X branch helps reduce classification errors. Future work could further optimize the model structure and features to improve discrimination capabilities for these categories.

### 5.2 Classification results confusion matrix diagram

**Fig 7:**
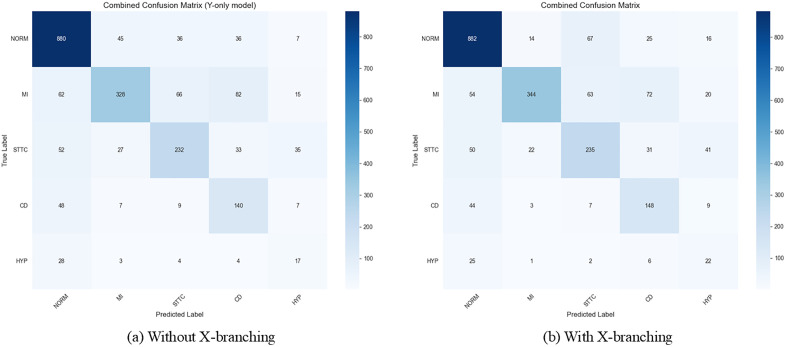
Confusion matrices for the MAF-Net model (with and without the X branch) across five hyperclasses on the PTB-XL dataset.

The confusion matrices depicted in Fig 7 provide a detailed representation of performance, illustrating the frequency with which the model accurately identifies each CVD and thereby highlighting their recall and error rates. The collection of confusion matrices generated by the MAF-Net model on the PTB-XL dataset.

Fig 7(a) shows the Y-only model without the X branch. In Fig 7(b), the model with the X branch demonstrates more accurate predictions for the NORM (normal) category compared to the left panel. The introduction of the X branch improves the model’s overall performance, particularly in predicting NORM (Normal), CD (Conduction Disturbance), and HYP (Ventricular Hypertrophy) categories. Despite this improvement, errors persist in the MI (Myocardial Infarction) and STTC (ST-T Segment Changes) categories. This may stem from the high similarity of features between these two categories and others, making accurate differentiation challenging for the model.

### 5.3 Bar chart comparison of classification results

**Fig 8:**
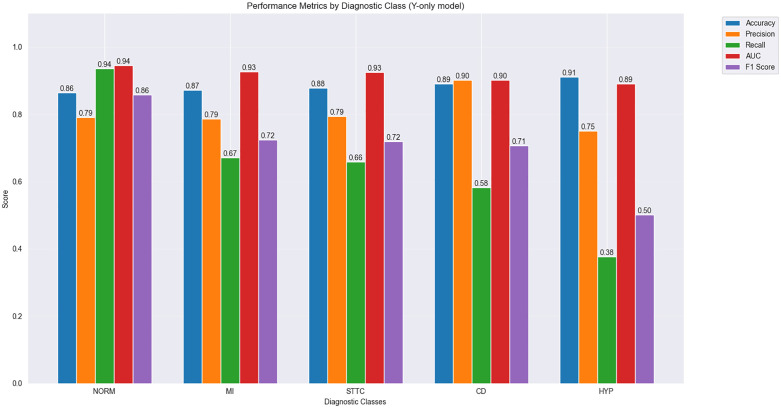
Bar chart comparing different metrics across five hyperclasses for the MAF-Net model (without X branch) on the PTB-XL dataset.

**Fig 9:**
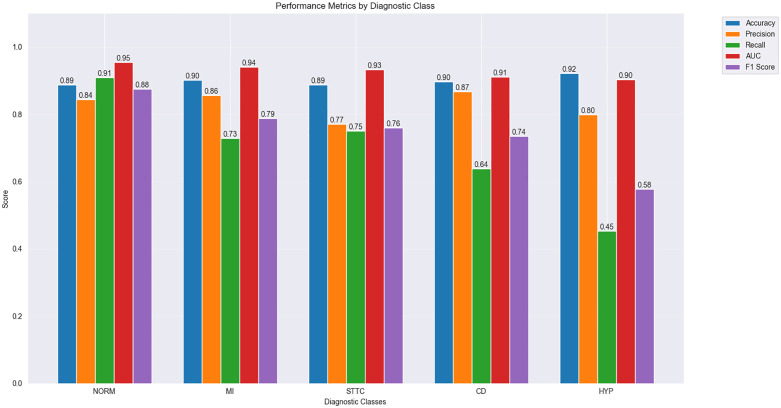
Bar chart comparing different metrics across five hyperclasses for the MAF-Net model (with X-branch) on the PTB-XL dataset.

Figs 8 and 9 present the model’s performance metrics across different diagnostic categories with and without the X branch, respectively. Each figure details five key performance indicators: Accuracy, Precision, Recall, AUC (Area Under the Curve), and F1 Score.

Fig 8 displays the classification performance of the model without the X branch: The NORM (Normal) category performed best with an accuracy of 0.86, recall of 0.94, and AUC of 0.94; The MI (Myocardial Infarction) category showed slightly lower recall and F1 score at 0.67 and 0.72, respectively;The STTC (ST-T segment changes) category exhibits lower precision and recall at 0.79 and 0.66, respectively; the CD (conduction block) category has a slightly lower AUC of 0.90; the HYP (ventricular hypertrophy) category shows lower precision and recall at 0.75 and 0.38, respectively.

Fig 9 demonstrates the performance of the MAF-Net model incorporating the X branch: The NORM (normal) category continues to exhibit optimal performance, with accuracy improved to 0.89, recall at 0.91, and AUC at 0.95; Performance in the MI (myocardial infarction) category has improved, with recall and F1 score increasing to 0.73 and 0.79, respectively;The STTC (ST-T segment changes) category shows improved performance, with precision and recall increasing to 0.77 and 0.75, respectively; the CD (conduction block) category achieves an AUC of 0.91; the HYP (ventricular hypertrophy) category demonstrates enhanced performance, with precision and recall increasing to 0.80 and 0.45, respectively.

Combining the analyses from Figs 8 and 9, the introduction of the X branch improved model performance across all categories, particularly in MI (myocardial infarction), STTC (ST-T segment changes), CD (conduction block), and HYP (ventricular hypertrophy). This demonstrates that the X branch has a significant positive impact on model performance through its extraction of clinically relevant data features.

### 5.4 t-SNE visualization of classification results

**Fig 10:**
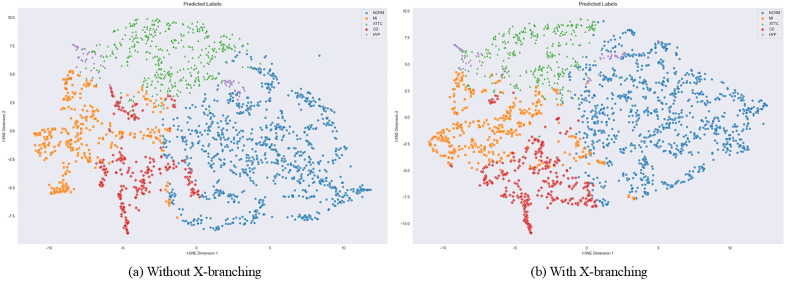
t-SNE visualization of spatial clustering for five hypercategories on the PTB-XL dataset using the MAF-Net model (with and without the X branch).

To illustrate the impact of the X branch’s clinical data features on CVD diagnosis within the PTB-XL dataset, a more intuitive visualization of the five-class classification results was achieved using the t-distributed stochastic neighbor embedding (t-SNE) algorithm. t-SNE is a widely used high-dimensional data visualization technique that maps high-dimensional data onto two- or three-dimensional spaces to facilitate observation of data distribution and clustering patterns.As shown in Fig 10, the colors of the points represent different types of cardiovascular diseases: NORM (Normal): (Blue), MI (Myocardial Infarction): (Orange), STTC (ST-T Segment Changes): (Green), CD (Conduction Disturbance): (Red), HYP (Hypertrophy): (Purple). The color distribution reflects the model’s classification results for the data.

It can be observed that in Fig 10(a) (model without X branch), data points from different categories exhibit some distribution overlap in the two-dimensional space, indicating that the model may have limited discrimination capability for certain categories. This is particularly evident in the MI (myocardial infarction) and STTC (ST-T segment changes) categories, whose distribution regions show significant overlap. This suggests the model may face challenges in distinguishing between these two categories.In contrast, the MAF-Net model incorporating the X branch (Fig 10(b)) exhibits clearer separation of data points across categories compared to the left panel, with reduced overlapping regions. This indicates that introducing the X branch enhances the model’s ability to distinguish categories, particularly for MI and STTC, whose distribution areas become more distinct.Distributions for other categories like NORM (normal), CD (conduction disturbance), and HYP (ventricular hypertrophy) also become more concentrated, indicating improved accuracy in recognizing these categories. Comparing the two t-SNE visualizations demonstrates the enhanced model performance after introducing the X branch while also highlighting residual errors in certain categories, providing direction for further model optimization.

### 5.5 This study has several limitations

First, model training and evaluation relied solely on the PTB-XL dataset, lacking external validation on other independent datasets or real clinical settings, which may impact the model’s generalization ability. Second, although preliminary interpretability analysis was conducted, there remains room for improvement in achieving full transparency of the model’s decision-making process. Finally, the types and number of clinical features were relatively limited; incorporating more diverse clinical indicators (e.g., lipid levels, blood pressure) could further enhance performance in future studies.

## 6. Conclusion

This paper proposes the MAF-Net model, a fusion network based on multimodal cross-attention, for cardiovascular disease classification. The model innovatively integrates clinical data features with electrocardiogram (ECG) data features from patients. The core novelty lies in introducing a bidirectional cross-attention fusion mechanism, which enables dynamic, bidirectional information exchange between clinical data and ECG signals, rather than simple feature concatenation.

Experimental results demonstrate that compared to existing state-of-the-art models, MAF-Net achieves the current best classification performance on the PTB-XL dataset, with an average AUC of 0.9407 ± 0.002 and an accuracy of 90.75% ± 0.32%. Ablation experiments further validate the effectiveness of the multimodal fusion strategy. This model provides an accurate and effective solution for the automated diagnosis of cardiovascular diseases.

Future work may further expand the model’s application scope, such as conducting external validation on datasets encompassing more diverse populations and exploring integration into clinical workflows for real-time prediction. Additionally, in-depth studies in clinical settings should further evaluate the method’s performance and reliability in automated cardiovascular disease diagnosis to fully unlock its potential application value.
